# New-Onset Autoantibody-Negative Diabetes With DKA Following Lorlatinib Therapy for ALK-Positive NSCLC

**DOI:** 10.1155/crie/8034190

**Published:** 2025-11-20

**Authors:** Ashni Dharia, Anna Pleet, Brittany West, Gayatri Jaiswal

**Affiliations:** ^1^Department of Internal Medicine, Allegheny General Hospital, Allegheny Health Network, 320 E North Avenue, Pittsburgh, Pennsylvania, USA; ^2^Department of Endocrinology, Allegheny General Hospital, Allegheny Health Network, 320 E North Avenue, Pittsburgh, Pennsylvania, USA

## Abstract

Lorlatinib, a tyrosine kinase inhibitor used in anaplastic lymphoma kinase (ALK)-positive non-small cell lung cancer (NSCLC), is associated with adverse events, including hyperglycemia. We present a case of a 52-year-old male with stage IV NSCLC and brain metastases who developed diabetic ketoacidosis (DKA) following lorlatinib initiation. The patient, without prior diabetes, presented with hyperglycemia (792 mg/dL), metabolic acidosis, and ketonuria 2 months after starting lorlatinib. He was treated for DKA and subsequently transitioned to a basal-bolus insulin regimen. Lorlatinib was withheld transiently and restarted once acute symptoms resolved. This case, along with three reported cases of lorlatinib-induced hyperglycemia, highlights a rare but serious potential complication. The mechanism of lorlatinib-induced hyperglycemia is unclear but may involve reduced insulin secretion. This case underscores the importance of monitoring for hyperglycemia in patients receiving lorlatinib, even in the absence of pre-existing diabetes, to enable early detection and prevent life-threatening complications like DKA.

## 1. Introduction

Lung cancer is the second most common cancer worldwide, with non-small cell lung cancer (NSCLC) accounting for the vast majority (~85%) of cases [1]. Considering the global burden of these cancers, the development of advanced targeted therapeutics has been paramount. In recent decades, the discovery of driver mutations has enabled drug development targeting specific molecular pathways unique to tumors. Lorlatinib is an antineoplastic, third-generation anaplastic lymphoma kinase (ALK)/ROS1 tyrosine kinase inhibitor that has demonstrated clinical benefit in treating advanced NSCLC, particularly in patients with central nervous system involvement or resistance to earlier ALK inhibitors [2, 3]. During the CROWN trial, lorlatinib was shown to be associated with longer progression-free survival and a higher frequency of intracranial response to brain metastasis when compared to crizotinib—the first-line agent used for advanced ALK-positive NSCLC [4]. However, lorlatinib's metabolic toxicity profile has raised concerns for patient safety and increased risk of hyperlipidemias.

It is well-recognized that targeted therapies increase the risk of dyslipidemias, such as hypercholesterolemia and hypertriglyceridemia [4]. The most common adverse events associated with lorlatinib noted in the CROWN trial are hyperlipidemia, edema, weight gain, peripheral neuropathy, hyperglycemia, and cognitive decline [4, 5]. The CROWN trial reported no cases of lorlatinib-associated diabetic ketoacidosis (DKA); however, DKA has emerged as an additional, potentially life-threatening complication [2, 3]. Here, we describe a case of lorlatinib-induced DKA in a patient with ALK-positive NSCLC and no prior history of diabetes mellitus.

## 2. Case Presentation

A 52-year-old male with a medical history of coronary artery disease, hypertension, and hyperlipidemia presented to his primary care provider in mid-January 2025 with a persistent dry cough that had been ongoing since the previous summer. He reported some symptomatic relief with steroids, inhalers, and antibiotics, but the cough never fully resolved. He also endorsed night sweats and an unintentional 40-pound weight loss over the past year. A chest X-ray obtained at the initial visit revealed multiple bilateral pulmonary nodules, along with an area of consolidation in the right upper lobe. A CT scan of the patient's chest, abdomen, and pelvis showed innumerable pulmonary nodules and masses and mediastinal lymphadenopathy that were concerning for metastatic disease. The largest mass was found in the right upper lobe, measuring 10.2 cm by 7.7 mm.

The patient was referred to Interventional Pulmonology, where he underwent ultrasound-guided fine-needle aspiration of the right supraclavicular lymph node 5 days after presentation. Oncology subsequently ordered a PET scan and a brain MRI to identify the cancer stage. The MRI revealed brain metastases in the right frontal lobe (measuring 1.9 cm) and in the left cerebellum (measuring 0.9 cm). The PET scan confirmed the presence of these pulmonary and intracranial lesions and showed no evidence of additional metastatic disease in the patient's abdomen or pelvis. Pathology from the lymph node biopsy confirmed a diagnosis of NSCLC with positive thyroid transcription factor-1 immunostaining. Molecular testing performed through Guardant360 identified an echinoderm microtubule-associated protein-like 4 (EML4) ALK fusion. Additional tissue analysis conducted via CARIS demonstrated ALK positivity, with 100% of tumor cells staining positive for IHC 3.

After confirming a diagnosis of stage IV NSCLC with lung and brain metastasis, the patient was prescribed 100 mg of lorlatinib daily. He reported improvement after initiating therapy. Because he remained neurologically asymptomatic, radiation oncology elected to defer gamma knife radiosurgery at that time. Approximately 1 month after initiating lorlatinib, the patient reported new symptoms of polyuria, polydipsia, blurred vision, slurred speech, and a feeling of unsteadiness that had been present for approximately 3 days. His random blood glucose was found to be 447 mg/dL (reference range 70–99 mg/dL), raising concern for lorlatinib-induced hyperglycemia. He was advised to temporarily discontinue lorlatinib and to resume at a reduced dose of 75 mg daily once his blood glucose levels dropped below 250 mg/dL or his symptoms improved to grade 1 severity.

Despite these recommendations, the patient resumed lorlatinib at the original 100 mg dose after a 3-day drug holiday. He soon presented to the emergency department with worsening symptoms, including progressive difficulty ambulating, slurred speech, blurred vision, generalized malaise, persistent polyuria, and polydipsia. On examination, he was hemodynamically stable but exhibited mild-to-moderate dysarthria and expressive aphasia.

The patient's initial laboratory studies revealed a serum sodium level of 124 mmol/L (reference range 136–145 mmol/L), blood glucose level of 792 mg/dL (reference range 70–99 mg/dL), an anion gap level of 18 mmol/L (reference range 7–16 mmol/L), bicarbonate level of 21 mmol/L (reference range 22–30 mmol/L), and β-hydroxybutyrate level of 2.61 mmol/L (reference range 0.02–0.30 mmol/L). His hemoglobin A1c was significantly elevated at 10.9% (reference range < 5.7%). Urinalysis was notable for 4+ glucose and 1+ ketones. Blood cultures drawn at admission remained negative at 48 h. A noncontrast head CT showed no acute intracranial hemorrhage or mass effect and demonstrated improved vasogenic edema in the right frontal lobe and left cerebellum compared to prior MRI imaging from 2 months prior.

**Figure 1:**
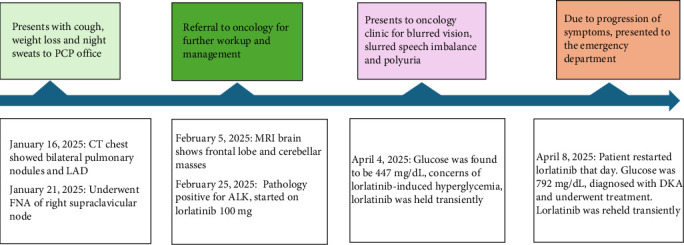
Timeline of key events from diagnosis of anaplastic lymphoma kinase (ALK)-positive non-small cell lung cancer (NSCLC) to development of lorlatinib-induced diabetic ketoacidosis (DKA). FNA, fine needle aspiration; LAD, lymphadenopathy; PCP, primary care provider.

Based on the clinical course and laboratory test results, the patient was diagnosed with lorlatinib-induced diabetes mellitus complicated by DKA. He had no prior history of diabetes, and his hemoglobin A1C level from 2023 was 5.5 %. Additionally, his test results were negative for insulinoma-associated-2 antigen and glutamic acid decarboxylase 65 antibodies. The clinical timeline of lorlatinib initiation and the subsequent development of DKA can be seen in Figure 1.

To treat the DKA, the patient was initiated on a continuous intravenous insulin infusion at a rate of 4–5 units/h. His blood glucose levels were monitored hourly, with insulin rates adjusted accordingly. Supportive management included aggressive, intravenous fluid resuscitation and electrolyte correction. It took ~12 h for DKA resolution. Endocrinology was consulted, given the new diagnosis of diabetes mellitus, and the patient was then transitioned to continuous subcutaneous insulin therapy consisting of a basal dose of 15 units/day and prandial doses of 9 units along with a sliding scale.

Medical oncology was reconsulted, and they recommended that lorlatinib be held at discharge. The patient was discharged in stable condition on 15 units of insulin glargine daily and insulin lispro with meals based on a sliding scale, with outpatient follow-up scheduled with endocrinology. At the time of discharge, his symptoms of blurred vision, slurred speech, and gait instability had resolved. At his oncology follow-up visit in mid-April 2025, his fasting blood glucose level was 170 mg/dL, and lorlatinib was restarted at a reduced dose of 75 mg daily. He was advised to monitor his blood glucose, and a continuous glucometer was placed as he continued insulin therapy. A restaging CT scan of the patient's chest, abdomen, and pelvis performed 3 days after his oncology follow-up, showed decreased size of the right upper lobe mass and bilateral pulmonary nodules, with no evidence of new or residual metastatic disease in the abdomen or pelvis. The outpatient laboratory test results showed a C-peptide level of 1.7 ng/mL (reference range 1.1–5.0 ng/mL) with a glucose level of 239 mg/dL (reference range 70–99 mg/dL); however, interpretation is challenging due to ongoing insulin therapy. The patient's high glucose levels are not improving with Glargine and Metformin. Recent fasting glucose levels are above 250 mg/dL. Oncology has temporarily stopped Lorlatinib treatment again, awaiting improved glucose readings.

## 3. Discussion

In our case report, we describe a case of lorlatinib-induced hyperglycemia and DKA in a patient with ALK-positive NSCLC with no prior history of diabetes mellitus and who was found to be antibody-negative. These findings underscore the need for close glycemic monitoring in patients undergoing lorlatinib treatment and other immune checkpoint inhibitors or targeted cancer treatments, including both those with and those without pre-existing diabetes. Lorlatinib is effective against ALK-positive NSCLC, and the most common ALK fusion partner is EML4 [5]. More than 15 different EML4:ALK fusion variants have been identified. Lorlatinib is effective against these variants, including those that have developed resistance to other ALK inhibitors like crizotinib [5]. Additionally, this highlights a metabolic toxicity that may stand as a potential barrier to maintaining optimal dosing and cancer control, especially in the event that the therapy must be paused or discontinued altogether.

There are currently three cases reported in the literature that have highlighted hyperglycemia induced by lorlatinib therapy, with two resulting in DKA [2, 3, 6, 7]. In 2021, Nakano et al. [6] reported that a patient with ALK-positive NSCLC and controlled type 2 diabetes mellitus experienced profound hyperglycemia 92 days after starting lorlatinib. Lorlatinib therapy continued without interruption, and glycemic control was eventually achieved with up-titration of the insulin regimen and oral hypoglycemics [6]. In 2025, Yanagisawa et al. [2, 3] described a patient with ALK-positive NSCLC and controlled type 2 diabetes mellitus that developed DKA 3 months after initiating lorlatinib; this patient was notably absent of islet cell autoantibodies. That patient temporarily paused lorlatinib treatment, but it was later resumed at a reduced dose with positive outcomes, and glycemic control was achieved by initiating insulin therapy [2, 3]. The authors proposed that insulin resistance, compounded by severe lorlatinib-induced hypertriglyceridemia, led to impaired glucose utilization, increased lipolysis, and ultimately ketoacidosis [2, 3]. Both of these cases involved patients with pre-existing type 2 diabetes [2, 3, 6]. Conversely, a 2023 abstract described a patient with ALK-positive paraspinal neuroblastoma and no prior history of diabetes, who developed DKA 14 months after starting lorlatinib [7]. This patient was started on outpatient insulin therapy, and lorlatinib was not reintroduced after this incident [7]. Additionally, a pharmacovigilance analysis using the FDA Adverse Events Reporting System found that lorlatinib was linked to a significant signal for both hyperglycemia and DKA [8]. According to the FDA label, hyperglycemia was reported in 9% of patients receiving lorlatinib, with grade 3 or 4 severity in 3.2% [9].

The exact mechanism of lorlatinib causing hyperglycemia is currently unclear; however, it is known that the ALK gene is part of the insulin receptor protein-tyrosine kinase receptor super family and can structurally mimic insulin receptors. It is speculated that inhibiting the ALK gene can lead to decreased insulin secretion, leading to hyperglycemia. Another mechanism could be related to lorlatinib inhibiting the insulin-like growth factor-1 receptor, which is involved in glucose metabolism [10]. This inhibition may further contribute to glucose dysregulation. In addition, the high penetration of lorlatinib across the blood–brain barrier may affect insulin receptors in the brain that regulate systemic glucose metabolism [2]. The pathophysiology in antibody-negative cases, such as the patient described in this report, appears to differ from typical autoimmune-mediated mechanisms. There is currently no published evidence that lorlatinib exerts direct cytotoxic or autoimmune effects on pancreatic β-cells. Instead, the prevailing hypothesis suggests that the drug's impact on lipid metabolism and body weight (hypercholesterolemia, hypertriglyceridemia, and weight gain) exacerbates insulin resistance and impaired glucose regulation, potentially precipitating DKA in metabolically vulnerable individuals [2, 3, 9].

In our above-described case, the GAD65 and IA-2 autoantibodies were negative, suggesting that this is unlikely related to immune-mediated destruction of pancreatic beta cells.

In terms of management of hyperglycemia/DKA from Lorlatinib therapy, there are limited cases to come up with conclusive recommendations. Review of case reports (Table 1), in two cases, Lorlatinib was discontinued. In one case, it was restarted at a lower dose, and glycemic control was managed with insulin therapy. In our case, when Lorlatinib was reintroduced, glucose levels rose. Since our patient declined prandial insulin, only basal insulin was continued along with metformin. Lorlatinib was on hold again because of this reason by the oncology team.

Based on review of case reports and our experience, our authors recommend that oncologists be aware of this important side effect of Lorlatinib and check a baseline hemoglobin A1c and fasting glucose before starting therapy. Monitoring with fasting glucose may also be important during therapy. Patients may be made aware of potential symptoms of hyperglycemia, such as polyuria, polydipsia. In terms of whether LorlatiNib should be restarted after developing hyperglycemia or DKA should be a multidisciplinary discussion. Patient preference is also important, keeping in mind that insulin initiation will likely be needed. The novel action of Lorlatinib and the critical indication of the medication in metastatic malignancy is an important consideration before discontinuing therapy.

## 4. Conclusion

Lorlatinib is an effective treatment for ALK-positive NSCLC, but it is associated with potential adverse metabolic effects, including hyperglycemia, insulin resistance, and DKA in rare cases. To date, the exact driving mechanisms of this process, particularly in autoantibody-negative cases, are not well understood. While DKA is a rare complication, this case highlights the importance of vigilance, early detection, and prompt management. Further research is needed to better understand and prevent lorlatinib-induced hyperglycemia and its potentially life-threatening consequences.

## Figures and Tables

**Table 1:** Summary of case reports of hyperglycemia/diabetes ketoacidosis (DKA) following initiation of lorlatinib.

Study	Malignancy	Known diabetes mellitus history, HbA1C prior to lorlatinib	Onset of hyperglycemic event/DKA after starting lorlatinib	HbA1C (mmol/mol) at time of hyperglycemia/DKA onset	Insulin regimen at discharge	Lorlatinib interruption/reintroduction
Nakano, 2022 [6]	ALK^+^ lung adenocarcinoma	Yes, 6.4%, on vildagliptin	92 days	16.1	18 units long-acting insulin and 70 units rapid-acting insulin	No
Sumal, 2023 [7]	ALK^+^ paraspinal neuroblastoma	No	14 months	14.8	54 units long-acting insulin and 48 units rapid-acting insulin	Yes, not reintroduced
Yanagisawa et al. [2, 3]	ALK^+^ lung adenocarcinoma	Yes, 6.0% on pioglitazone and teneligliptin	3 months	11.5	15 units long-acting insulin and 52 units rapid-acting insulin	Yes, 2 weeks later at dose of 75 mg/day

*Note:* This case had lorlatinib-induced hyperglycemia without DKA.

Abbreviations: ALK, anaplastic lymphoma kinase; DM, diabetes mellitus.

## Data Availability

Data sharing is not applicable to this article as no new data were created or analyzed in this study.
